# Novel Loci Controlling Parasite Load in Organs of Mice Infected With *Leishmania major*, Their Interactions and Sex Influence

**DOI:** 10.3389/fimmu.2019.01083

**Published:** 2019-06-07

**Authors:** Tatyana Kobets, Marie Čepičková, Valeriya Volkova, Yahya Sohrabi, Helena Havelková, Milena Svobodová, Peter Demant, Marie Lipoldová

**Affiliations:** ^1^Laboratory of Molecular and Cellular Immunology, Institute of Molecular Genetics, Academy of Sciences of the Czech Republic, Prague, Czechia; ^2^Faculty of Science, Charles University, Prague, Czechia; ^3^Roswell Park Comprehensive Cancer Center, Buffalo, NY, United States

**Keywords:** *Leishmania major*, visceral leishmaniasis, parasite load, PCR-ELISA, susceptibility to Infection, QTL, mouse model, sex influence

## Abstract

Leishmaniasis is a serious health problem in many countries, and continues expanding to new geographic areas including Europe and USA. This disease, caused by parasites of *Leishmania* spp. and transmitted by phlebotomine sand flies, causes up to 1.3 million new cases each year and despite efforts toward its functional dissection and treatment it causes 20–50 thousands deaths annually. Dependence of susceptibility to leishmaniasis on sex and host's genes was observed in humans and in mouse models. Several laboratories defined in mice a number of *Lmr* (*Leishmania major response*) genetic loci that control functional and pathological components of the response to and outcome of *L. major* infection. However, the development of its most aggressive form, visceral leishmaniasis, which is lethal if untreated, is not yet understood. Visceral leishmaniasis is caused by infection and inflammation of internal organs. Therefore, we analyzed the genetics of parasite load, spread to internal organs, and ensuing visceral pathology. Using a new PCR-based method of quantification of parasites in tissues we describe a network-like set of interacting genetic loci that control parasite load in different organs. Quantification of *Leishmania* parasites in lymph nodes, spleen and liver from infected F_2_ hybrids between BALB/c and recombinant congenic strains CcS-9 and CcS-16 allowed us to map two novel parasite load controlling *Leishmania major response* loci, *Lmr24* and *Lmr27*. We also detected parasite-controlling role of the previously described loci *Lmr4, Lmr11, Lmr13, Lmr14, Lmr15*, and *Lmr25*, and describe 8 genetic interactions between them. *Lmr14, Lmr15, Lmr25*, and *Lmr27* controlled parasite load in liver and lymph nodes. In addition, *Leishmania* burden in lymph nodes but not liver was influenced by *Lmr4* and *Lmr24*. In spleen, parasite load was controlled by *Lmr11* and *Lmr13*. We detected a strong effect of sex on some of these genes. We also mapped additional genes controlling splenomegaly and hepatomegaly. This resulted in a systematized insight into genetic control of spread and load of *Leishmania* parasites and visceral pathology in the mammalian organism.

## Introduction

Leishmaniasis is a neglected tropical disease, which belongs to the top health problems because it is endemic in 98 countries in Asia, Africa, the Americas, and the Mediterranean region (1–3) and is gradually expanding to new areas, including Central Europe and USA (2, 4–9). The disease occurs in cutaneous, mucocutaneous, and visceral forms (9). It is caused by the protozoan intracellular parasite *Leishmania* transmitted by *Phlebotomus* spp. in the Old World and *Lutzomyia* spp. in the New World. The parasite can infect about 70 species of vertebrates, including humans (10–13). In addition, there are specific groups of asymptomatic infection (14), and post-kala-azar dermal leishmaniasis (15). Visceral leishmaniasis is fatal in more than 95% of cases if left untreated (9). Up to 1.3 million new cases occur annually: 300 000 are visceral and 1 million are cutaneous and mucocutaneous and about 20–50 thousands patients die (13). In the infected mammalian organism, *Leishmania* parasites invade “professional phagocytes,” including monocytes, macrophages, and neutrophils and can also reside in dendritic cells (DC) (16), immature myeloid precursor cells, hepatocytes, and fibroblasts; the parasite can also enter sialoadhesin-positive stromal macrophages (17).

Treatment of leishmaniasis is difficult because of the lack of reliable drugs. Existing leishmanicidal agents show severe side effects. In addition, the treatment is costly and not readily available to a majority of patients. In spite of numerous attempts to develop vaccination against leishmaniasis, there are still no safe and effective vaccines suitable for humans (18, 19). Clinical form and susceptibility to leishmaniasis are dependent on parasite species, environmental and social factors, and also on nutrition and genotype of the host (3, 10, 16, 20).

Parasite load is one of the most important parameters of leishmaniasis determining the course of infection and the degree of susceptibility. However, the information about genetic control of parasite load remains incomplete and fragmented; there is no systematic description of the control of parasite load in combination with other pathological parameters and influence of sex on these genes is not known for any of the studied *Leishmania* species. The use of mouse models in studies of selected candidate genes and also for hypothesis-generating genome-wide association and linkage analysis, revealed several genes and loci controlling parasite burden (16, 21–23) (Table 1). However, quantification of parasites had been a laborious task providing inaccurate results due to technical problems. These problems were reduced with development of sensitive PCR-based assays (18), which permitted to perform genome wide-search (21, 23).

**Table 1:** Genetic control of parasite load in mouse leishmaniasis.

**Parasite**	**Organ**	**Gene/Locus**	**References**
*L. donovani*	Spleen	*Slc11a1* (weak effect), *H2, Lyst*	(24, 25)
	Liver	*Slc11a1, H2, Ir2*	(25–28)
	Bone marrow	*H2*	(25)
*L. mexicana*	Spleen	*Slc11a1, H2*	(29)
	Liver	*Slc11a1, H2*	(29)
*L. infantum*	Spleen	*Slc11a1, H2*	(25)
	Liver	*Slc11a1, H2*	(25)
	Bone marrow	*H2*	(25)
*L. major*	Spleen	*Lmr5*	(21)
	Skin	*Dice1.2*	(30)
	Lymph nodes	*Lmr20*	(21)
*L. tropica*	Spleen	*Ltr3, Ltr6*	(23)
	Liver	*Ltr2, Ltr4, Ltr8*	(23)
	Lymph nodes	*Ltr1, Ltr4*	(23)

*Leishmania species and strains used in described experiments: (21), L. major (LV 561 (MHOM/IL/67/LRC-L137 JERICHO II); (23), L. tropica (MHOM/1999/TR/SU23); (24), L. donovani 2S (MHOM/SD/61/2S); (25), L. infantum, zymodem MON-1, L. donovani L82 (LV9) (MHOM/ET/67/L82); (26), L. donovani L82 (LV9) (MHOM/ET/67/L82); (27), L. donovani L82 (LV9) (MHOM/ET/67/L82); (28), L. donovani, 3S (MHOM/SD/62/3S); (29), L. mexicana (MNYC/B2/62/M379); (30) L. major (WHOM/IR/-/173)*.

We explored genetic control of parasite load in different organs after *L. major* infection. This parasite is the predominant causative agent of human cutaneous leishmaniasis in the Old World, in rare cases it can visceralize in an immunocompromised (HIV infected) host (31), but *L. major* strain (MRHOM/IR/75/ER) was described to visceralize also in an immunocompetent individual (32). Instances of *L. major* visceralization in non-immunocompromised people may suggest the presence of genetic factors determining extreme forms of high susceptibility to *L. major* infection. Infection by *L. major* in mouse is controlled by multiple genes. These multiple genes-loci have been mapped in three different resistant strains—C57BL/10Sn (B10.D2), C57BL/6 and STS—using the susceptible strain BALB/c for mapping in each case (16, 22); in cross with B10.D2 using for infection *L. major* (strain WHOM/IR/-/173), in crosses with C57BL/6 and STS *L. major* V121 (the cloned line V121 derived from MHOM/IL/67/Jericho II) and *L. major* strain (LV 561 (MHOM/IL/67/LRC-L137 JERICHO II), respectively. These experiments revealed 26 *Lmr* (*Leishmania major* response) and 5 *Lmrq* (*Leishmania major* resistance QTL) loci that determine skin lesion size, splenomegaly, hepatomegaly, cytokine levels in blood serum, eosinophil infiltration into lymphatic nodes, and parasite numbers in different organs (16, 21, 22, 33). Several loci (*Lmr4*/*Lmrq1, Lmr5*/*Lmrq3, Lmr6*/*Lmrq4*, and *Lmr12*/*Dice1b*) detected in crosses with resistant strains STS and B10.D2 overlap, which might indicate in these loci not only general response across different mouse strains, but also general response to different *L. major* strains.

In the present study, we tested parasite load and dissemination in F_2_ hybrids of BALB/c and recombinant congenic mouse strains CcS-9 and CcS-16, infected by *L. major* LV561. The disease development during previous experiments showed no significant difference between females and males of the CcS-16 strain after infection with *L. major* (34); however the influence of sex was present in CcS-9 strain (33). These differences determined selection of sex of mice used in the present F_2_ hybrid study. The current study aims to provide a first systematic a genome wide search description of the genetic control of parasite load in mammalian organs after *L. major* infection.

## Materials and Methods

### Mice

Recombinant congenic (RC) strains of mice of the BALB/c-c-STS/Dem (CcS/Dem) series, containing random distinct segments of 12.5% STS/A (STS) genes on the background of BALB/cHeA (BALB/c) genome (35), exhibit various susceptibility to *Leishmania* infection and proved to be a powerful tool in research of genetic control of the disease (16, 21, 23, 33, 36, 37). BALB/c is a standard inbred mouse strain, STS is an inbred mouse strain of Swiss origin. The parts of CcS-9 and CcS-16 genomes inherited from the BALB/c or STS parents were defined (35).

At the time of experiments, mice of CcS-9 strain were in the 40 generation of inbreeding, and therefore highly homozygous. F_2_ hybrids between CcS-9 and BALB/c (age 11–21 weeks at the time of infection, mean and median age 14.8 and 15 weeks, respectively) were produced at the Institute of Molecular Genetics; 254 F_2_ hybrids between BALB/c and CcS-9 comprised 139 females and 115 males. Mice were tested in three independent experimental groups; male and female mice were placed into separate rooms, and males were caged individually.

In CcS-16 experiments, only females were used due to absence of sex differences in previous experiments. Mice of CcS-16 strain were in the generation 37 of inbreeding, and therefore highly homozygous. We produced 577 female F_2_ hybrids between CcS-16 and BALB/c (age at the time of infection, 14–17 weeks) and tested them in four independent experiments.

Mice were kept in the animal facility of Institute of Molecular Genetics AS CR.

### Parasites

*Leishmania major* LV 561 (MHOM/IL/67/LRC-L137 JERICHO II) was maintained in rump lesions of BALB/c females. Amastigotes were transformed to promastigotes using SNB-9 (38); 10^7^ promastigotes from 6 days old subculture 2 were inoculated in 50 μl sterile saline s.c. into mouse rump (39).

### Disease Phenotype

The size of the primary skin lesions was measured weekly using a Vernier caliper gauge. Mice were euthanized 8 weeks after infection and body, spleen, and liver weights were recorded. The blood, spleen, liver, and inguinal lymph nodes were collected for the further analysis. Splenomegaly (enlargement of the spleen) and hepatomegaly (enlargement of the liver) were calculated as organ-to-body weight ratio × 1000. Parasite load was presented in relative units as concentration of parasite DNA in ng per 1 μl.

### Cytokine and IgE Levels

IgE, IL-4, IL-10, IL-12, IL-13, and IFNγ levels in serum were determined using the primary and secondary monoclonal antibodies (IgE: R35-72, R35-118, IL-4: 11B11, BVD6-24G2, IL-10: JES5-2A5, JES5-16E3, IL-12: C15.6, C17.8, IFNγ: R4-6A2, XMG1.2) and standards from Pharmingen (San Diego, CA, USA) (purified mIgE: C38-2, recombinant mouse IL-4, mIL-10, mIL-12 p70 heterodimer, and mIFNγ). The enzyme-linked immunosorbent assay (ELISA) was performed as recommended by Pharmingen. IL-13 level in serum were determined using the Murine IL-13 ELISA Development Kit 900-K207 (by PeproTech EC (London, United Kingdom), which contained both primary and secondary monoclonal antibodies and standard and the ELISA was performed as recommended by PeproTech EC. The IL-4, IL-10, IL-12, IL-13, IFNγ, and IgE levels were estimated using the curve fitter program KIM-E (Schoeller Pharma, Prague, Czech Republic).

### Genotyping of F_2_ Hybrids

The frozen archive material of F_2_ crosses genotyped in previous mapping experiments (33, 40) was used for measurement of parasite DNA and analysis of genetic linkage.

In CcS-9 experiment, STS derived segments were typed in F_2_ hybrids using 19 microsatellite markers on eight chromosomes: D2Mit148, D2Mit283, D4Mit7, D4Mit17, D4Mit23, D4Mit53, D4Mit172, D5Mit24, D5Mit143, D6Mit122, D6Mit274, D9Mit15, D11Mit141, D11Mit242, D11Nds10, D11Nds18, D16Mit19, D17Mit120, and D17Mit122 as described elsewhere (33).

In CcS-16 experiment, the segments of STS origin on 9 chromosomes were typed in F_2_ hybrids using 23 markers: D2Mit51, D2Mit102, D2Mit156, D2Mit283, D2Mit389, D2Nds3, D3Mit11, D3Mit25, D4Mit153, D6Mit48, D6Mit320, D10Mit67, D10Mit103, D11Mit37, D11Mit139, D11Mit242, D16Mit126, D17Mit38, D17Mit130, D18Mit35, D18Mit40, D18Mit49, and D18Mit120 as described in Vladimirov et al. (40).

### Measurement of Parasite Load in Organs

Total DNA was isolated from frozen lymph nodes, spleen, and liver samples, and parasite load was measured using PCR-ELISA according to the previously published protocol (41). Briefly, total DNA was isolated using a standard proteinase procedure (42). For detection of *Leishmania* parasite DNA in total DNA, PCR was performed using two primers (digoxigenin-labeled F 5′-ATT TTA CAC CAA CCC CCA GTT-3′ and biotin-labeled R 5′-GTG GGG GAG GGG CGT TCT-3′ (VBC Genomics Biosciences Research, Austria). The 120-bp fragment within the conserved region of the kinetoplast minicircle of *Leishmania* parasite was amplified. In each PCR reaction, 50 ng of extracted total DNA was used. As a positive control, 20 ng of *L. major* DNA per reaction was amplified as a highest concentration of the standard. In CcS-9 experiment, 26-cycle PCR reaction was used for quantification of parasites in lymph nodes and spleen, and 35 cycles for liver. In CcS-16 experiment, 24-cycle PCR reaction was used for quantification of parasites in lymph nodes; 28 cycles for spleen, and 33 cycles for liver. Parasite load was determined by measurement of the PCR product with the modified ELISA protocol (Pharmingen, San Diego, USA). Concentration of *Leishmania* DNA was measured at the ELISA Reader Tecan with the curve fitter program KIM-E (Schoeller Pharma, Prague, Czech Republic) using least squares-based linear regression analysis (21, 41).

### Statistical Analysis

The role of genetic factors in control of parasite dissemination was estimated with ANOVA using Statistical for Windows 12.0 (StatSoft, Inc., Tulsa, OK, USA). Markers and interactions with *P* < 0.05 were combined in a single comparison. Genotype (marker), sex and age were fixed factors and the experiment was random factor. The time course of skin lesion development was evaluated on the basis of weekly measurements of lesion size in each mouse in weeks 4–8 after infection. Variance components and mixed model ANOVA of Statistica for Windows 12.0 (StatSoft, Inc., Tulsa, OK, USA) with marker as the fixed variable and the week of observation as the covariate have been used to evaluate the linkage.

When necessary, the original values of an analyzed parameter were transformed for normalization of the distribution as described in the legends to the Tables. For whole genome significance values (corrected *P*-values), the observed *P*-values (αT) were adjusted using the following formula (43):

$$\begin{array}{l} \alpha {\text{T}}^{*}\approx \left[\text{C}+2\rho \text{Gh}\left(\text{T}\right)\right]\alpha \text{T} \end{array}$$

In the present formula, G = 1.75 Morgan (the length of the segregating part of the genome: 12.5% of 14 M); C = 8 (number of chromosomes segregating in cross between CcS-9 and BALB/c) or C = 9 (between CcS-16 and BALB/c); ρ = 1.5 for F_2_ hybrids; h(T) = the observed statistic (F ratio).

The percent of the phenotypic variance explained by a certain locus or an interaction between loci was calculated subtracting the sums of squares of the model without this variable from the sum of squares of the full model and this difference, divided by the total regression sums of squares:

$$\begin{array}{l} \frac{\left(\text{SS}(\text{b1},\text{b2},\text{b3},\text{b4|b0}))-(\text{SS}(\text{b1},\text{b2},\text{b3},\text{b4},\text{b5|b0})\right)}{\left(\text{RSS}(\text{b1},\text{b2},\text{b3},\text{b4},\text{b5|b0}\right)} \end{array}$$

## Results

The present studies revealed two novel *Lmr* loci, new functions of six previously mapped *Lmr* loci and described multiple and heterogeneous genetic effects influencing parasite dissemination into the host organism. Because the STS-derived regions in CcS-9 and CcS-16 are different, we detected in their respective F_2_ hybrids with BALB/c different loci controlling parasite load.

### The CcS-9 Strain: Two Novel *Lmr* Loci, Multiple Interactions, and Sex Dependent Control of Parasite Load

**Figure 1:**
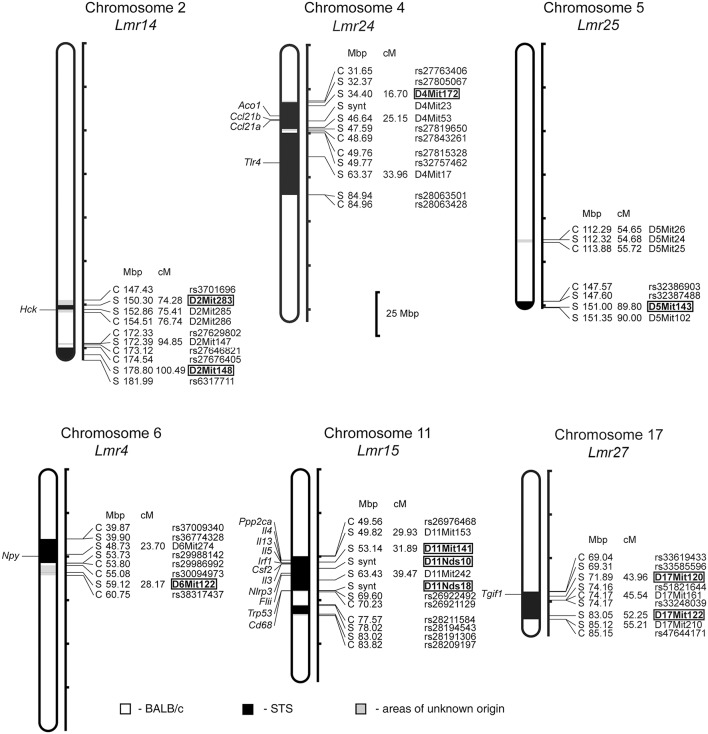
Positions of the loci controlling parasite load in organs of the RC strain CcS-9. The regions derived from the BALB/c strain are indicated as white; the regions of the STS origin are indicated as black; and the areas of undetermined origin are gray. Only the markers that determine the boundaries between BALB/c and STS-derived segments, and the markers used for statistical calculations are shown. Boxes indicate the markers that exhibited significant *P*-values, corrected for genome-wide search. The genes that participate in the infection-related immune processes (16, 17, 20, 22, 33) and are located within the present *Lmr* loci are listed next to their positions at the chromosomes. Abbreviations indicate genes related to the response to *Leishmania* ssp.: *Hck*, hemopoietic cell kinase; *Aco1*, aconitase 1; *Ccl21a*, chemokine (C-C motif) ligand 21A (serine); *Ccl21b*, chemokine (C-C motif) ligand 21B (leucine), *Tlr4*, toll-like receptor 4; *Npy*, neuropeptide Y; *Ppp2ca*, protein phosphatase 2, formerly 2A; catalytic subunit, alpha isoform; *Il3*, interleukin 3; *Il4*, interleukin 4; *Il13*, interleukin 13; *Irf1*, interferon regulatory factor 1; *Csf2*, colony stimulating factor 2 (granulocyte-macrophage); *Nlrp3*, NLR family, pyrin domain containing 3; *Flii*, flightless I actin binding protein; *Trp53*, transformation related protein 53; Cd68, CD68 antigen; *Tgif1*, TGFB-induced factor homeobox 1.

In F_2_ hybrids prepared from the parental strain BALB/c and the RC strain CcS-9, the analysis of parasite load in inguinal lymph nodes, spleen and liver followed by linkage analysis, revealed both main effect loci and interactions of genes located on chromosomes 2, 4, 5, 6, 11, and 17. Two novel *Lmr* loci, *Lmr24*, and *Lmr27* were detected in the CcS-9 experiments (Figure 1).

Both in males and females, a homozygous STS allele (SS) at the *Lmr14* linked to the marker D2Mit283 determined the highest parasite load in inguinal lymph nodes interacting with homozygous STS alleles (SS) of the *Lmr25* linked to D5Mit143 (corrected *P* = 0.0314, 2.1% of variance explained), and also with the *Lmr24* linked to D4Mit172 (corrected *P* = 0.0247, 2% of variance explained) (Table 2).

**Table 2:** Interactions that control parasite load in lymph nodes of CcS-9 derived F_2_ hybrids of both sexes.

**Lymph nodes**		**D5Mit143 (*****Lmr25)***											
		**CC**			**CS**			**SS**			***P*-value**	**corr. *P***	**% of explained variance**
D2Mit283 (*Lmr14*)	CC	*n =* 20	**2.34**	5.45 ± 0.22	*n =* 21	**1.33**	4.89 ± 0.21	*n =* 18	**1.20**	4.79 ± 0.23	0.00044	**0.0314**	**2.1**
	CS	*n =* 31	**1.03**	4.64 ± 0.19	*n =* 57	**2.19**	5.39 ± 0.13	*n =* 30	**1.93**	5.27 ± 0.18			
	SS	*n =* 24	**1.09**	4.69 ± 0.20	*n =* 32	**1.60**	5.08 ± 0.17	*n =* 20	**2.26**	5.42 ± 0.21			
**Lymph nodes**		**D2Mit283 (*****Lmr14*****)**											
		**CC**			**CS**			**SS**			***P*****-value**	**corr**. ***P***	**% of explained variance**
D4Mit172 (*Lmr24*)	CC	*n =* 15	**1.25**	4.83 ± 0.27	*n =* 22	**1.78**	5.18 ± 0.21	*n =* 16	**1.70**	5.14 ± 0.24	0.00034	**0.0247**	**2.0**
	CS	*n =* 28	**1.47**	4.99 ± 0.18	*n =* 59	**2.05**	5.32 ± 0.12	*n =* 36	**1.01**	4.61 ± 0.17			
	SS	*n =* 16	**2.03**	5.31 ± 0.23	*n =* 37	**1.20**	4.79 ± 0.18	*n =* 24	**2.29**	5.43 ± 0.20			

*Means, standard error of mean (SE) and P-values were calculated by analysis of variance. The letters C and S indicate the BALB/c or STS allele, respectively; n indicates the number of mice in each group. Transformed means ± SE are shown next to non-transformed mean values in bold. The values of concentration of parasite DNA (ng/ml) in total DNA isolated from lymph nodes were transformed to obtain normal distribution: a natural logarithm of (value × 100). Only P-values significant after correction for genome-wide significance are shown*.

Genetic control of parasite load in lymph nodes was strongly dependent on sex (Tables 3, 4): in interactions of sex with markers D11Nds10, D11Nds18, D11Mit141, D2Mit148, D17Mit122 - *P* < 10^∧(−30)^; D11Nds10 and D17Mit122, as well as D6Mit122 and D17Mit122 - *P* < 10^(∧−30)^. In females, the highest parasite load in lymph nodes were observed in mice homozygous for BALB/c (CC) allele in the *Lmr15* linked to D11Nds10 (corrected *P* = 0.000696, 7.42% of variance explained) (Table 3A). In males, a homozygous STS allele (SS) of the *Lmr14* linked to D2Mit148 (corrected *P* = 0.031, 10.5% of variance explained), and the novel locus *Lmr27* linked to D17Mit122 (corrected *P* = 0.012, 15.3% of variance explained) determined higher parasite load in inguinal lymph nodes (Table 3B). Homozygotes in SS allele on the novel locus *Lmr27* linked to D17Mit122 determined the highest parasite load in males in two interactions: with CC allele of the *Lmr4* linked to D6Mit122 (corrected *P* = 0.00427, 7.4% of variance explained), and CS allele on the *Lmr15* linked to D11Nds10 (corrected *P* = 0.0261, 6% of variance explained), respectively (Table 4).

**Table 3A:** The *Lmr15* controls parasite load in lymph nodes of CcS-9 derived females.

**Lymph nodes**	**CC**			**CS**			**SS**			
**Marker**	***n***	**Mean**	**Transformed mean** **± SE**	***n***	**Mean**	**Transformed mean** **± SE**	***n***	**Mean**	**Transformed mean** **± SE**	***P*-value**	**corr. *P***	**% of Explained variance**
D11Mit141 (*Lmr15*) both sexes	56	**2.14**	5.37 ± 0.15	127	**1.56**	5.05 ± 0.10	70	**1.11**	4.71 ± 0.13	0.00419	0.157	2.56
D11Mit141 (*Lmr15*) females	36	**1.44**	4.97 ± 0.17	69	**1.02**	4.62 ± 0.13	33	**0.45**	3.80 ± 0.18	0.000014	**0.00102**	**6.38**
D11Mit141 (*Lmr15*) males	20	**3.18**	5.76 ± 0.23	58	**3.58**	5.88 ± 0.14	37	**2.99**	5.70 ± 0.17	0.695	6.890	0.29
D11Nds10 (*Lmr15*) both sexes	58	**2.22**	5.40 ± 0.16	129	**1.54**	5.03 ± 0.09	66	**1.13**	4.73 ± 0.13	0.00395	0.149	3.01
D11Nds10 (*Lmr15*) females	36	**1.58**	5.06 ± 0.18	70	**0.99**	4.60 ± 0.13	32	**0.45**	3.80 ± 0.18	0.0000092	**0.000696**	**7.42**
D11Nds10 males	22	**3.12**	5.74 ± 0.26	59	**3.11**	5.74 ± 0.15	34	**3.37**	5.82 ± 0.19	0.935	7.811	2.5
D11Nds18 (*Lmr15*) both sexes	51	**1.67**	5.12 ± 0.16	136	**1.79**	5.19 ± 0.08	66	**1.08**	4.69 ± 0.12	0.00281	0.112	2.95
D11Nds18 (*Lmr15*) females	33	**1.21**	4.80 ± 0.16	73	**0.97**	4.58 ± 0.11	32	**0.50**	3.91 ± 0.16	0.00018	**0.0101**	**4.2**
D11Nds18 (*Lmr15*) males	18	**1.86**	5.23 ± 0.37	63	**2.61**	5.56 ± 0.19	34	**4.13**	6.02 ± 0.2	0.000175	**0.0106**	**16.7**

*Means, standard error of mean (SE) and P-values were calculated by analysis of variance. The numbers in bold give the average non-transformed values. The letters C and S indicate the BALB/c or STS allele, respectively; n indicates the number of mice in each group. The values of concentration of parasite DNA (ng/ml) in total DNA isolated from lymph nodes were transformed to obtain normal distribution: a natural logarithm of (value × 100). Only P-values significant after correction for genome-wide significance are shown*.

**Table 3B:** The *Lmr14* and *Lmr27* control parasite load in lymph nodes of CcS-9 derived males.

**Lymph nodes**	**CC**			**CS**			**SS**			
**Marker**	***n***	**Mean**	**Transformed mean ± SE**	***n***	**Mean**	**Transformed mean ± SE**	***n***	**Mean**	**Transformed mean ± SE**	***P* value**	**corr. *P***	**% of explained variance**
D2Mit148 (*Lmr14*) both sexes	78	**1.72**	5.15 ± 0.13	121	**1.61**	5.08 ± 0.11	54	**1.68**	5.13 ± 0.15	0.908	7.725	0.93
D2Mit148 (*Lmr14*) females	43	**0.83**	4.41 ± 0.14	69	**0. 89**	4.49 ± 0.12	26	**0. 67**	4.20 ± 0.18	0.411	5.220	0.39
D2Mit148 (*Lmr14*) males	35	**3.04**	5.72 ± 0.19	52	**3.11**	5.74 ± 0.14	28	**16.20**	7.39 ± 0.39	0.000616	**0.03095**	**10.5**
D17Mit122 (*Lmr27*) both sexes	74	**1.61**	5.08 ± 0.12	142	**1.42**	4.96 ± 0.10	37	**2.04**	5.32 ± 0.19	0.209	3.399	2.28
D17Mit122 (*Lmr27*) females	42	**0.89**	4.49 ± 0.15	77	**0.77**	4.35 ± 0.11	19	**0.89**	4.48 ± 0.21	0.678	6.813	0.17
D17Mit122 (*Lmr27*) males	32	**3.44**	5.84 ± 0.18	65	**2.84**	5.65 ± 0.13	18	**15.56**	7.35 ± 0.36	0.000206	**0.0118**	**15.3**

*Means, standard error of mean (SE) and P-values were calculated by analysis of variance. The numbers in bold give the average non-transformed values. The letters C and S indicate the BALB/c or STS allele, respectively; n indicates the number of mice in each group. The values of concentration of parasite DNA (ng/ml) in total DNA isolated from lymph nodes were transformed to obtain normal distribution: a natural logarithm of (value × 100). Only P-values significant after correction for genome-wide significance are shown*.

**Table 4:** Interactions that control parasite load in lymph nodes of CcS-9 derived males.

**Lymph nodes**		**D11Nds10 (*****Lmr15*****)**											
		**CC**			**CS**			**SS**			***P* value**	**corr. *P***	**% of explained variance**
D17Mit122 (*Lmr27*)	CC	*n =* 7	**2.50**	5.52 ± 0.31	*n =* 12	**3.22**	5.77 ± 0.24	*n =* 13	**5.06**	6.23 ± 0.23	0.000339	**0.0261**	**6.0**
	CS	*n =* 11	**3.97**	5.98 ± 0.25	*n =* 37	**2.92**	5.68 ± 0.14	*n =* 17	**2.00**	5.30 ± 0.20			
	SS	*n =* 4	**4.68**	6.15 ± 0.41	*n =* 10	**52.10**	8.56 ± 0.26	*n =* 4	**15.53**	7.35 ± 0.41			
**Lymph nodes**		**D6Mit122 (*****Lmr4*****)**											
		**CC**			**CS**			**SS**			***P*****-value**	**corr**. ***P***	**% of explained variance**
D17Mit122 (*Lmr27*)	CC	*n =* 10	**5.21**	6.26 ± 0.26	*n =* 13	**2.76**	5.62 ± 0.23	*n =* 9	**2.84**	5.65 ± 0.23	0.0000467	**0.00427**	**7.4**
	CS	*n =* 17	**2.35**	5.46 ± 0.20	*n =* 35	**4.26**	6.05 ± 0.14	*n =* 13	**2.31**	5.44 ± 0.20			
	SS	*n =* 5	**73.38**	8.90 ± 0.37	*n =* 9	**5.65**	6.34 ± 0.28	*n =* 4	**9.13**	6.82 ± 0.41			

*Means, standard error of mean (SE) and P-values were calculated by analysis of variance. The letters C and S indicate the BALB/c or STS allele, respectively; n indicates the number of mice in each group. Transformed means ± SE are shown next to non-transformed mean values in bold. The values of concentration of parasite DNA (ng/ml) in total DNA isolated from lymph nodes were transformed to obtain normal distribution: a natural logarithm of (value × 100). Only P-values significant after correction for genome-wide significance are shown*.

No sex differences were detected in control of parasite load in liver. Parasite load in liver were therefore calculated for total group (both males and females), and revealed the effect of the CC allele on *Lmr15* linked to D11Mit141 (corrected *P* = 0.0037, 8.6% of variance explained) and D11Nds10 (corrected *P* = 0.0011, 9.6% of variance explained) in control of higher parasite burden (Table 5). Parasites load in liver was also controlled by an interaction between the novel locus *Lmr27* linked to D17Mit120 and *Lmr14* linked to D2Mit283 (corrected *P* = 0.0184, 6.9% of variance explained) (Table 6) and by an interaction of *Lmr27* linked to D17Mit122 with *Lmr25* linked to D5Mit143 (suggestive linkage; corrected *P* = 0.0640, 6.2% of variance explained). Lowest parasite load are linked with the interaction of homozygotes in STS (SS) allele at *Lmr25* with the homozygotes in BALB/c (CC) allele at *Lmr27*.

**Table 5:** The *Lmr15* controls parasite load in liver of CcS-9 derived F_2_ hybrids of both sexes.

**Liver**	**CC**			**CS**			**SS**			
**Marker**	***n***	**Mean**	**Transformed mean ± SE**	***n***	**Mean**	**Transformed mean ± SE**	***n***	**Mean**	**Transformed mean ± SE**	***P*-value**	**corr. *P***	**% of explained variance**
D11Mit141 (*Lmr15*)	56	**3.43**	5.84 ± 0.11	128	**2.90**	5.67 ± 0.07	69	**1.87**	5.23 ± 0.10	0.000061	**0.00373**	**8.6**
D11Nds10 (*Lmr15*)	58	**3.36**	5.82 ± 0.12	130	**3.06**	5.72 ± 0.08	65	**1.87**	5.23 ± 0.10	0.000015	**0.00106**	**9.6**

*Means, standard error of mean (SE) and P-values were calculated by analysis of variance. The numbers in bold give the average non-transformed values. The letters C and S indicate the BALB/c or STS allele, respectively; n indicates the number of mice in each group. The values of concentration of parasite DNA (ng/ml) in total DNA isolated from liver were transformed to obtain normal distribution: a natural logarithm of (value × 100). Only P-values significant after correction for genome-wide significance are shown*.

**Table 6:** Interactions that control parasite load in liver of CcS-9 derived F_2_ hybrids of both sexes.

**Liver**		**D2Mit283 (*****Lmr14*****)**											
		**CC**			**CS**			**SS**			***P* value**	**corr. *P***	**% of explained variance**
D17Mit120 (*Lmr27*)	CC	*n =* 17	**1.99**	5.29 ± 0.19	*n =* 29	**2.75**	5.62 ± 0.15	*n =* 27	**3.29**	5.80 ± 0.15			
	CS	*n =* 31	**3.56**	5.87 ± 0.14	*n =* 63	**3.08**	5.73 ± 0.10	*n =* 44	**1.78**	5.18 ± 0.12	0.00024	**0.0184**	**6.9**
	SS	*n =* 12	**1.91**	5.25 ± 0.22	*n =* 25	**2.67**	5.59 ± 0.15	*n =* 5	**3.44**	5.84 ± 0.35			
**Liver**		**D5Mit143 (*****Lmr25*****)**											
		**CC**			**CS**			**SS**			***P*****-value**	**corr**. ***P***	**% of explained variance**
	CC	*n =* 30	**3.17**	5.76 ± 0.13	*n =* 28	**3.93**	5.97 ± 0.14	*n =* 16	**1.57**	5.05 ± 0.19			
D17Mit122 (*Lmr27*)	CS	*n =* 36	**2.80**	5.63 ± 0.13	*n =* 64	**2.98**	5.70 ± 0.10	*n =* 41	**2.54**	5.54 ± 0.12	0.00096	**0.0640**	**6.2**
	SS	*n =* 9	**2.21**	5.40 ± 0.26	*n =* 18	**2.02**	5.31 ± 0.20	*n =* 11	**3.85**	5.95 ± 0.23			

*Means, standard error of mean (SE) and P-values were calculated by analysis of variance. The letters C and S indicate the BALB/c or STS allele, respectively; n indicates the number of mice in each group. Transformed means ± SE are shown next to non-transformed mean values in bold. The values of concentration of parasite DNA (ng/ml) in total DNA isolated from liver were transformed to obtain normal distribution: a natural logarithm of (value × 100). Only P-values significant after correction for genome-wide significance are shown*.

No loci that determine parasite load in spleen were found in F_2_ hybrids derived from the CcS-9 strain.

### Loci Controlling Organ Pathology and Immune Response in CcS-9

Analysis of different parameters of the disease indicated a novel *Lmr* locus which controls organ pathology and systemic immune response (Table 7). *Lmr24* on chromosome 4 influenced splenomegaly, levels of IFNγ, and IL-4 in serum and development of skin lesions. Control of splenomegaly was linked with the marker D4Mit23 (corrected *P* = 0.0426, 4.76% of variance explained), skin lesion size (kinetics of development of skin lesions)—with D4Mit172 and D4Mit23 (corrected *P* = 0.00201 and 0.0226, respectively), level of IFNγ in serum—with D4Mit53 (corrected *P* = 0.000692, 8.08% of variance explained), and level of IL-4 in serum—with D4Mit53 (corrected *P* = 0.0132, 5.72% of variance explained). The STS allele of these markers was responsible for lower level of IFNγ and IL-4 in serum, but with larger splenomegaly and larger skin lesions.

**Table 7:** Loci controlling organ pathology and immunological parameters in *Leishmania major*-infected F2 hybrids between CcS-9 and BALB/c.

**Phenotype**	**Locus**	**Marker**	**Genotype**						***P*-value**	**corr. *P***	**% of explained variance**
			**CC**		**CS**		**SS**				
Splenomegaly	*Lmr15*	D11Mit141	**17.86** ± 0.74 (*n =* 56)		**17.29** ± 0.42 (*n =* 128)		**14.86** ± 0.57 (*n =* 70)		0.00066	**0.0313**	**9.16**
		D11Nds10	**18.07** ± 0.73 (*n =* 58)		**17.18** ± 0.42 (*n =* 130)		**14.77** ± 0.59 (*n =* 66)		0.00047	**0.0235**	**11.80**
	*Lmr24*	D4Mit23	**15.71** ± 0.64 (*n =* 57)		**15.83** ± 0.45 (*n =* 122)		**18.17** ± 0.54 (*n =* 75)		0.00093	**0.0426**	**4.76**
Hepatomegaly	*Lmr15*	D11Nds18	**64.02** ± 1.03 (*n =* 51)		**63.05** ± 0.60 (*n =* 136)		**58.50** ± 0.90 (*n =* 66)		0.000029	**0.00191**	**9.76**
Lesion size (in 7th week after infection)	*Lmr15*	D11Mit141	**83.68** ± 4.92 (*n =* 56)		**69.72** ± 3.00 (*n =* 128)		**55.68** ± 4.23 (*n =* 70)		0.000095	**0.00555**	**8.64**
		D11Mit242	**84.81** ± 5.00 (*n =* 51)		**69.18** ± 2.85 (*n =* 139)		**56.23** ± 4.00 (*n =* 64)		0.000044	**0.00274**	**7.55**
		D11Nds18	**90.07** ± 5.55 (*n =* 51)		**69.47** ± 2.88 (*n =* 137)		**55.87** ± 3.93 (*n =* 66)		0.0000035	**0.000269**	**10.06**
		D11Nds10	**86.07** ± 4.64 (*n =* 58)		**68.63** ± 2.91 (*n =* 130)		**55.25** ± 4.36 (*n =* 66)		0.000010	**0.000722**	**12.04**
Lesion size (in 8th week after infection)	*Lmr15*	D11Nds10	**101.56** ± 5.62 (*n =* 58)		**88.47** ± 3.25 (*n =* 130)		**73.30** ± 4.95 (*n =* 66)		0.00058	**0.0282**	**9.37**
		D11Nds18	**100.93** ± 5.11 (*n =* 51)		**87.94** ± 3.18 (*n =* 137)		**76.31** ± 4.36 (*n =* 66)		0.00093	**0.0424**	**4.21**
Lesion size (kinetics)	*Lmr15*	D11Nds18	**32.77**	5.72 ± 0.13 (*n =* 51)	**28.37**	5.33 ± 0.08 (*n =* 137)	**25.54**	5.05 ± 0.11 (*n =* 66)	0.00060	**0.0282**	–
		D11Nds10	**31.33**	5.60 ± 0.12 (*n =* 58)	**29.09**	5.39 ± 0.08 (*n =* 130)	**24.91**	4.99 ± 0.11 (*n =* 66)	0.00090	**0.0406**	–
	*Lmr24*	D4Mit172	**27.19**	5.21 ± 0.13 (*n =* 53)	**26.40**	5.14 ± 0.08 (*n =* 124)	**32.92**	5.74 ± 0.11 (*n =* 77)	0.000032	**0.00201**	–
		D4Mit23	**26.97**	5.19 ± 0.12 (*n =* 57)	**26.87**	5.18 ± 0.08 (*n =* 122)	**32.38**	5.69 ± 0.11 (*n =* 75)	0.00047	**0.0226**	–
Level of IgE in serum	*Lmr15*	D11Mit141	**35.09**	3.12 ± 0.10 (*n =* 56)	**25.26**	2.81 ± 0.07 (*n =* 127)	**15.29**	2.39 ± 0.09 (*n =* 70)	0.00000052	**0.0000460**	**10.93**
		D11Mit242	**33.89**	3.09 ± 0.11 (*n =* 51)	**25.78**	2.83 ± 0.07 (*n =* 138)	**17.70**	2.51 ± 0.10 (*n =* 64)	0.00028	**0.0148**	**6.23**
		D11Nds10	**36.94**	3.17 ± 0.11 (*n =* 58)	**25.96**	2.84 ± 0.07 (*n =* 129)	**13.72**	2.31 ± 0.11 (*n =* 66)	0.00000013	**0.0000129**	**13.00**
Level of INFγ in serum	*Lmr24*	D4Mit53	**13.20**	2.81 ± 0.14 (*n =* 59)	**5.93**	2.04 ± 0.09 (*n =* 120)	**6.58**	2.12 ± 0.12 (*n =* 74)	0.0000097	**0.000692**	**8.08**
Level of IL-4 in serum	*Lmr24*	D4Mit53	**1.79**	0.47 ± 0.06 (*n =* 59)	**1.20**	0.17 ± 0.05 (*n =* 120)	**1.26**	0.21 ± 0.06 (*n =* 74)	0.00025	**0.0132**	**5.72**
		D11Nds10	**1.65**	0.42 ± 0.07 (*n =* 58)	**1.48**	0.34 ± 0.04 (*n =* 129)	**1.10**	0.09 ± 0.06 (*n =* 66)	0.00046	**0.0230**	**6.73**
Level of IL-13 in serum	*Lmr14*	D2Mit283	**518.66**	22.77 ± 1.18 (*n =* 44)	**551.08**	23.48 ± 0.84 (*n =* 82)	**830.30**	28.81 ± 1.24 (*n =* 57)	0.00027	**0.0146**	**10.76**

*Only linkages significant at whole genome level are given. In order to obtain normal distribution required for analysis of variance, the following transformations were used: lesion size (mm^2^)—power of 0.5; IgE in serum (μg/ml)—power of 0.32; IFN-γ in serum (ng/ml)—power of 0.4; IL-4 in serum (ng/ml)—Box-Cox transformation: [(x^∧^(−0.720642))−1]/(−0.720642), IL-13 in serum (pg/ml)—power of 0.5. The numbers in bold give the average non-transformed values. C and S indicate the presence of BALB/c and STS allele, respectively; n indicates number of mice*.

In addition to the novel locus, we detected new functions of previously mapped loci (Table 7). *Lmr14* was detected in F_2_ hybrids between CcS-16 and BALB/c. It was previously shown that it controlled hepatomegaly, splenomegaly, level of IgE and IFNγ in serum, and also infiltration of eosinophils to inguinal lymph nodes (33, 40, 44, 45). The present study revealed the role of *Lmr14* (marker D2Mit283) in control of level of IL-13 in serum (corrected *P* = 0.0146, 10.76% of variance explained). The STS allele of this marker was linked with higher level of IL-13 in serum. *Lmr15* was detected in F_2_ hybrids between CcS-16 and BALB/c and it influences hepatomegaly, IFNγ level in serum and infiltration of eosinophils in inguinal lymph nodes (33, 40, 45). The present study revealed several additional effects of *Lmr15*: control of splenomegaly (markers D11Mit141 and D11Nds10, corrected *P* = 0.0313 and 0.0235, respectively, 9.16 and 11.80% of variance explained), lesion size in week 8 after infection (markers D11Nds10 and D11Nds18, corrected *P* = 0.0282 and 0.0424, respectively, 9.37 and 4.21% of variance explained), kinetics of development of skin lesions (markers D11Nds18 and D11Nds10, corrected *P* = 0.0282 and 0.0406, respectively) and level of IgE in serum (markers D11Mit141, D11Mit242, and D11Nds10, corrected *P* = 0.0000460, 0.0148 and 0.0000129, respectively, 10.93, 6.23, and 13.00% of variance explained). The STS allele of these markers was linked with lower level of IgE in serum and smaller splenomegaly and skin lesions.

In addition, we detected two interactions between loci which control different parameters of the infection (Table 8). Interaction between markers D11Nds10 (*Lmr15*) and D16Mit19 (*Lmr18*) controlled lesion size in 7th week after infection. BALB/c homozygosity at *Lmr15* synergizes with BALB/c homozygosity at *Lmr18* in enlarging size of skin lesions (corrected *P* = 0.044, 6.71% of variance explained). Interaction between marker D4Mit53 (*Lmr24*) and D6Mit122 (*Lmr4*) controlled level of IL-10 in serum. STS homozygosity at *Lmr24* synergized with STS homozygosity at *Lmr4* in increasing of level of IL-10 in serum (corrected *P* = 0.0156, 9.61% of variance explained. The control of organ pathology and immune response in CcS-9 was not sex dependent.

**Table 8:** Interactions between loci that control lesion size and level of IL-10 in serum in *Leishmania major*-infected F_2_ hybrids between CcS-9 and BALB/c.

**Lesion size in 7^**th**^ week after infection**		**D16Mit19 (*****Lmr18*****)**											
		**CC**			**CS**			**SS**			***P*-value**	**corr. *P***	**% of explained variance**
D11Nds10 (*Lmr15*)	CC	*n =* 99	111.45 ± 10.05	*n =* 30	73.89 ± 5.61	*n =* 19	66.25 ± 6.88	0.00064	**0.044**	**6.71**
	CS	*n =* 32	59.48 ± 5.32	*n =* 66	68.33 ± 3.75	*n =* 32	76.07 ± 5.40			
	SS	*n =* 20	63.07 ± 6.80	*n =* 36	55.56 ± 5.13	*n =* 10	51.56 ± 9.47			
**Level of IL-10 in serum**		**D4Mit53 (*****Lmr24*****)**											
		**CC**			**CS**			**SS**			***P*** **value**	**corr**. ***P***	**% of explained variance**
D6Mit122 (*Lmr4*)	CC	*n =* 11	**1.52**	0.32 ± 0.03	*n =* 24	**2.10**	0.47 ± 0.02	*n =* 15	**2.01**	0.45 ± 0.02	0.00020	**0.0156**	**9.61**
	CS	*n =* 27	**2.12**	0.47 ± 0.02	*n =* 44	**1.94**	0.43 ± 0.01	*n =* 31	**1.92**	0.43 ± 0.02			
	SS	*n =* 10	**1.87**	0.42 ± 0.03	*n =* 20	**1.93**	0.43 ± 0.02	*n =* 15	**2.15**	0.47 ± 0.02			

*In order to obtain normal distribution required for analysis of variance, the following transformation was used for level of IL-10 in serum (ng/ml) – [(x^∧^(−1.372544)]-1)/(−1.372544). C and S indicate the presence of BALB/c and STS allele, respectively; n indicates number of mice. Bold: non-transformed values*.

### The CcS-16 Strain: One Novel *Lmr* Locus Confirmed, Control of Parasite Load Detected for Two Additional Loci

**Figure 2:**
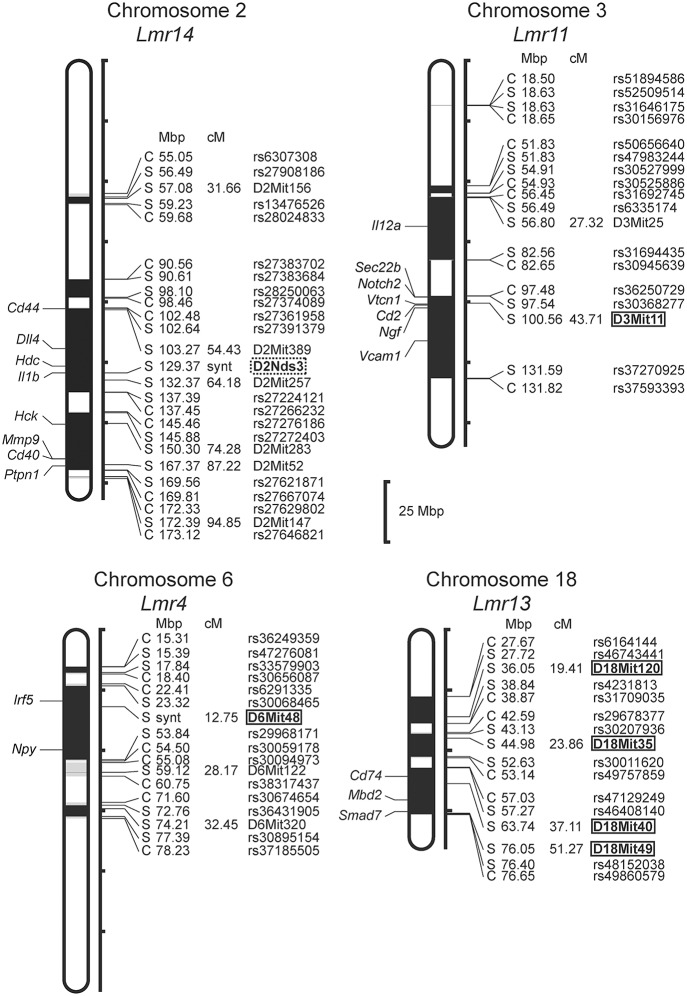
Positions of the loci controlling parasite load in organs of the RC strain CcS-16. The regions derived from the BALB/c strain are indicated as white; the regions of the STS origin are indicated as black; and the areas of undetermined origin are gray. Only the markers that determine the boundaries between BALB/c and STS-derived segments, and the markers used for statistical calculations are shown. Boxes indicate the markers that exhibited significant *P*-values, corrected for genome-wide search, dotted box—suggestive linkage. The genes that participate in the infection-related immune processes (16, 17, 20, 22, 23) and are located within the present *Lmr* loci are listed next to their positions at the chromosomes. Abbreviations indicate genes related to the response to *Leishmania* ssp.: *Cd44*, CD44 antigen; *Dll4*, delta like canonical Notch ligand 4; *Hdc*, histidine decarboxylase; *Il1b*, interleukin 1 beta; *Hck*, hemopoietic cell kinase; *Mmp9*, matrix metallopeptidase 9; *Cd40*, CD40 antigen; *Ptpn1*, protein tyrosine phosphatase, non-receptor type 1; *Il12a*, interleukin 12a; *Sec22b*, SEC22 homolog B, vesicle trafficking protein; *Notch2*, notch 2; *Vtcn1*, V-set domain containing T cell activation inhibitor 1; *Cd2*, CD2 antigen; *Ngf*, nerve growth factor; *Vcam1*, vascular cell adhesion molecule 1; *Irf5*, interferon regulatory factor 5; *Npy*, neuropeptide Y; *Cd74*, CD74 antigen [invariant polypeptide of major histocompatibility complex, class II antigen-associated)], *Mbd2*, methyl-CpG binding domain protein 2; *Smad7*, SMAD family member 7.

Only female mice were used in the study with F_2_ hybrids between the BALB/c parental mouse strain and the RC strain CcS-16, because previous analysis of response to *L. major* in different RC strains (34) showed no significant dependence on sex in CcS-16 mice (in contrast to the strain CcS-9 described above). We confirmed a presence of a novel parasite controlling cluster *Lmr4* (Figures 1, 2; Table 9) on chromosome 6, which influences parasite load in lymph nodes. A homozygous BALB/c (CC) allele in the *Lmr4* linked to D6Mit48 was responsible for higher parasite load (corrected *P* = 0.017, 4.95% of variance explained) (Table 9). The effect of *Lmr13* at the chromosome 18 linked to D18Mit40 on parasite load in liver was opposite to that of *Lmr4*: the STS allele (SS) was responsible for higher parasite load (corrected *P* = 0.00056, 10.4% of variance explained) (Table 9). Parasite load in liver controls also an interaction between *Lmr14* linked to D2Nds3 with *Lmr13* linked to D18Mit120 (suggestive linkage, corrected *P* = 0.0777, 4.8% of explained variance) (Table 10). Two genotype combinations determine highest parasite load: STS (SS) homozygotes in *Lmr13* with BALB/c (CC) homozygotes in *Lmr14*, and STS (SS) homozygotes in *Lmr14* with BALB/c (CC) homozygotes in *Lmr13*. A homozygous STS allele of *Lmr13* linked to the markers D18Mit35 (corrected *P* = 0.0073, 4.85% of variance explained) and D18Mit120 (corrected *P* = 0.019, 5.18% of variance explained) determined the higher load of parasites in spleen (Table 9). Parasite load in spleen was also controlled by *Lmr13* linked to D18Mit49 in interaction with *Lmr11* linked to D3Mit11 (corrected *P* = 0.0295, 3.4% of variance explained). Highest parasite load was observed in combination of STS (SS) homozygotes in *Lmr13* and BALB/c (CC) homozygotes in *Lmr11* (Table 10).

**Table 9:** The *Lmr4* controls parasite load in lymph nodes, and *Lmr13* controls parasite load in liver and spleen of CcS-16 derived F_2_ hybrids.

	**CC**			**CS**			**SS**					
**Marker**	***n***	**Mean**	**Transformed mean ± SE**	***n***	**Mean**	**Transformed mean ± SE**	***n***	**Mean**	**Transformed mean ± SE**	***P*-value**	**corr. *P***	**% of explained variance**
**Lymph nodes**												
D6Mit48 (*Lmr4*)	104	**1.98**	5.29 ± 0.12	230	**1.16**	4.75 ± 0.09	109	**1.12**	4.72 ± 0.12	0.00032	**0.017**	**4.95**
**Liver**												
D18Mit40 (*Lmr13*)	92	**0.52**	3.96 ± 0.12	178	**0.43**	3.75 ± 0.09	71	**1.03**	4.63 ± 0.16	0.0000078	**0.00056**	**10.4**
**Spleen**												
D18Mit35 (*Lmr13*)	136	**0.52**	3.95 ± 0.13	274	**0.87**	4.46 ± 0.09	133	**1.07**	4.67 ± 0.12	0.00013	**0.0073**	**4.85**
D18Mit120 (*Lmr13*)	133	**0.52**	3.95 ± 0.13	284	**0.87**	4.47 ± 0.09	130	**0.99**	4.59 ± 0.12	0.00038	**0.019**	**5.18**

Means, standard error of mean (SE) and P-values were calculated by analysis of variance. The letters C and S indicate the BALB/c or STS allele, respectively; n indicates the number of mice in each group. The numbers in bold give the average non-transformed values. The values of concentration of parasite DNA (ng/ml) in total DNA isolated from lymph nodes, liver and spleen were transformed to obtain normal distribution: a natural logarithm of a (value *100). Only P-values significant after correction for genome-wide significance are shown.

**Table 10:** Interactions that control parasite load in spleen and liver of CcS-16 derived F_2_ hybrids.

**Spleen**		**D3Mit11 (*****Lmr11*****)**											
		**CC**			**CS**			**SS**			***P*-value**	**corr. *P***	**% of explained variance**
D18Mit49 (*Lmr13*)	CC	*n =* 27	**0.40**	3.68 ± 0.25	*n =* 78	**0.93**	4.53 ± 0.14	*n =* 37	**0.63**	4.14 ± 0.21			
	CS	*n =* 59	**0.78**	4.35 ± 0.17	*n =* 175	**0.83**	4.42 ± 0.10	*n =* 48	**0.84**	4.43 ± 0.18	0.00042	**0.0295**	**3.4**
	SS	*n =* 35	**1.45**	4.98 ± 0.21	*n =* 53	**0.59**	4.07 ± 0.17	*n =* 34	**0.99**	4.59 ± 0.22			
**Liver**		**D2Nds3 (*****Lmr14*****)**											
		**CC**			**CS**			**SS**			***P*** **value**	**corr**. ***P***	**% of explained variance**
	CC	*n =* 28	**0.48**	3.87 ± 0.18	*n =* 37	**0.49**	3.88 ± 0.15	*n =* 15	**0.80**	4.38 ± 0.24			
D18Mit120 (*Lmr13*)	CS	*n =* 38	**0.37**	3.61 ± 0.15	*n =* 99	**0.45**	3.81 ± 0.10	*n =* 41	**0.53**	3.96 ± 0.15	0.0012	**0.0777**	**4.8**
	SS	*n =* 15	**0.97**	4.58 ± 0.24	*n =* 41	**0.77**	4.35 ± 0.15	*n =* 22	**0.37**	3.62 ± 0.20			

Means, standard error of mean (SE) and P-values were calculated by analysis of variance. The letters C and S indicate the BALB/c or STS allele, respectively; n indicates the number of mice in each group. Transformed means ± SE are shown next to mean non-transformed values in bold. The values of concentration of parasite DNA (ng/ml) in total DNA isolated from spleen and liver were transformed to obtain normal distribution: a natural logarithm of (a value*100). Only P-values significant after correction for genome-wide significance are shown.

## Discussion

Parasite load and dissemination in visceral organs of infected host belong to the most important parameters in leishmaniasis. Elucidation of host determinants of parasite control could help to better understand these critical disease mechanisms. We have therefore addressed the following questions: genetic control of parasite load in organs after *L. major* infection, analysis of relationship among parasite-controlling genes and the genes controlling organ pathology, and role of sex in control of parasite load. We defined two new genetic loci controlling parasite load (*Lmr24* and *Lmr27*) and described that six previously mapped loci (*Lmr4, Lmr11, Lmr13, Lmr14, Lmr15*, and *Lmr25*) also control parasite load. This enabled us to show a genetic network controlling interaction between parasite and mammalian host.

### Control of Parasite Load in Organs: Multiple Genes and Distinct Organ Specificities That Operate in a Network With Many Gene-Gene Interactions

**Figure 3:**
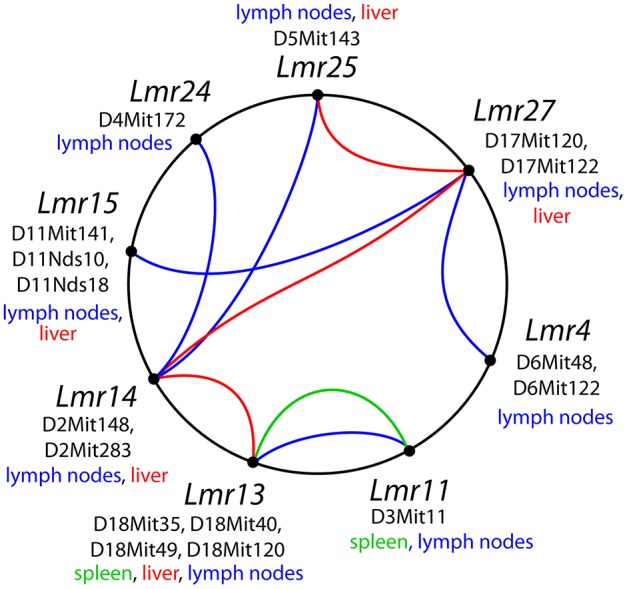
Interactions among loci controlling parasite load after *L. major* infection. The lines that connect related loci indicate interactions controlling a certain phenotype: parasite load in lymph nodes—blue color, in spleen—green, in liver—purple. Main gene effects of the *Lmr13, Lmr14, Lmr15, Lmr28*, and *Lmr29* are also indicated.

Visceral leishmaniasis is a life-threatening disease (9, 13) and hence understanding parasite spread to organs is essential. Although many of its host, vector, and parasite determinant have been described (46), the role of the host in control of parasite visceralization remains largely unknown. The importance of host determinants that can limit leishmaniasis to a cutaneous form or allow progressive visceral pathology is stressed by the observation that even though *L. major* causes mostly only cutaneous pathology in human, cases of visceralization are also known (31, 32). We have therefore studied parasite load after *L. major* infection in three organs: lymph nodes, spleen and liver, and found that parasite load in each tested organ is controlled by multiple genes. In this work, we report six loci (*Lmr4, Lmr14, Lmr15, Lmr24, Lmr25*, and *Lmr27*) that control parasite load in lymph nodes of the strains CcS-9 and CcS-16 (Table 11). *Lmr4, Lmr14*, and *Lmr15* can operate independently from other genes (main effect loci), whereas *Lmr24, Lmr25*, and *Lmr2*7 operate only in interaction with other loci (Figure 3). Previously, we described another locus, *Lmr20*, which influences parasite load in lymph nodes of the strain CcS-11 (21).

**Table 11A:** Summary of loci that control parasite load detected in CcS-9 study.

**Chr**.	***Lmr* loci**	**Markers**	**Sex**	**Parasite control in organ**	**Response to parasite**
2	*Lmr14*	D2Mit148			IL-13 in serum
		D2Mit283	M	Lymph nodes	Eosinophils in lymph nodes^**^^#^
2 and 4	Interaction, *Lmr14* and *Lmr24*	D2Mit283		Lymph nodes	
		D4Mit172			
2 and 5	Interaction, *Lmr14* and *Lmr25*	D2Mit283		Lymph nodes	
		D5Mit143			
2 and 17	Interaction, *Lmr14* and *Lmr27*	D2Mit283		Liver	
		D17Mit120			
4	*Lmr24*	D4Mit23			Skin lesion Splenomegaly IL-4, IFNγ in serum
		D4Mit53			
		D4Mit172			
4 and 6	Interaction, *Lmr4* and *Lmr24*	D4Mit53			IL-10 in serum
		D6Mit122			
5	*Lmr25*	D5Mit143			Eosinophils in lymph nodes^***^
5 and 17	Interaction, *Lmr25* and *Lmr27*	D5Mit143		Liver^*^	
		D17Mit122			
6 and 17	Interaction, *Lmr4* and *Lmr27*	D6Mit122	M	Lymph nodes	
		D17Mit122			
9	Interaction, *Lmr15* and *Lmr26*	D9Mit15 D11Nds10	M		Eosinophils in lymph nodes^***^
11	*Lmr15*	D11Nds18 D11Nds10 D11Mit141		Liver	Skin lesion Splenomegaly Hepatomegaly IL-4, IgE in serum
			F	Lymph nodes	
			M	Lymph nodes	
11 and 16	Interaction, *Lmr15* and *Lmr18*	D11Nds10 D16Mit19			Skin lesion
11 and 17	Interaction, *Lmr15* and *Lmr27*	D11Nds10 D17Mit122	M	Lymph nodes	
17	*Lmr27*	D17Mit122	M	Lymph nodes	

* *Suggestive linkage*.

** *Data from Slapničková et al. (33)*.

*** *Interaction between sex and marker was not significant*.

# *Significant only in males from the cross CcS-9 × BALB/c*.

**Table 11B:** Summary of loci that control parasite load and response to infection in CcS-16.

**Chr**.	***Lmr* loci**	**Markers**	**Parasite control in organ**	**Immune response**
2	*Lmr14*	D2Mit52 D2Mit102 D2Mit283 D2Mit389 D2MitNds3		Splenomegaly Hepatomegaly Spontaneous proliferation of splenocytes from infected mice IFNγ in serum
2 and 10	Interaction, *Lmr5*, and *Lmr14*	D2Mit102 D10Mit103 D2Mit389		IL-12, IgE in serum
2 and 16	Interaction, *Lmr12* and *Lmr14*	D2Mit283 D16Mit126		TNFα in serum
2 and 18	Interaction, *Lmr13* and *Lmr14*	D2Nds3 D18Mit120	Liver^*^	TNFα in serum
3 and 18	Interaction, *Lmr11* and *Lmr13*	D3Mit11 D18Mit49	Spleen	
6	*Lmr4*	D6Mit48	Lymph nodes	
10 and 16	Interaction, *Lmr12* and *Lmr19*	D10Mit65 D16Mit126		Spontaneous proliferation of splenocytes from infected mice
11	*Lmr15*	D11Mit37 D11Mit 139 D11Mit 242		Hepatomegaly IFNγ in serum
16	*Lmr12*	D16Mit126		IL-4, IgE in serum
18	*Lmr13*	D18Mit35 D18Mit40 D18Mit120	Spleen Liver	Lesion size IgE in serum

*Table summarizes data from Vladimirov et al. (40), Badalová et al. (44), and Havelková et al. (45) and this study*.

* *Suggestive linkage*.

We have found two loci that control parasite load in spleen of the strain CcS-16: *Lmr13* and *Lmr11* (Table 11B). *Lmr13* operates as main effect locus, role of *Lmr11* is visible only in interaction with *Lmr13* (Tables 9, 10). No locus controlling parasite load in spleen was detected in the strain CcS-9 although spleens of this strain contain parasites (47). This might be caused by the fact that parasite load in spleen in the strain CcS-9 is controlled by multiple weak loci and our experiments lacked the power to detect them. Thus, with the previously detected *Lmr5* controlling parasites in spleen in CcS-11 (21), there are three different loci controlling *L. major* load in spleen. Parasite load in liver is controlled by two main effect loci *Lmr13* and *Lmr15*, operating in the strains CcS-16 and CcS-9, respectively, and three loci *Lmr14, Lmr25* (suggestive linkage) and *Lmr27* (in the strain CcS-9), whose effect is observed only in interaction.

Comparison of loci that control parasite burden in organs indicates organ specific control of parasite load, where dissemination of parasites to lymph nodes, spleen and liver were controlled by distinct sets of loci that only partly overlaps (Table 11). Some loci, such as *Lmr13, Lmr14, Lmr15, Lmr25*, and *Lmr27* determined parasite load in two different organs, effects of others were limited to only one organ. Organ specific control was described also in candidate gene based studies of parasite burden after infection with *L. donovani*, where genes *Slc11a1, H2* (25) and *Lyst* (24) control parasite load in spleen, whereas *Slc11a1* (26), *H2* (27, 28) and *Ir2* (28) determine parasite load in liver. Genome-wide studies with *L. tropica* infection showed that parasite load in lymph nodes are determined by *Ltr1* and *Ltr4*, in spleen by *Ltr3* and *Ltr6*, and in liver by *Ltr2, Ltr4*, and *Ltr8* (23) (Table 1). On the mechanistic level, organ-specific genetic control might be based on organ-specific immune response to *Leishmania* parasites (48). Some of the loci that determine parasite load in *L. donovani* (MHOM/SD/62/3S) and *L. tropica* (MHOM/1999/TR/SU23) overlap with the *Lmr* loci: *Lmr13* that controls parasite load in liver and spleen co-localize with *Ltr8* influencing parasite load in liver (23), *Lmr14* (suggestive linkage) influencing in interaction with *Lmr13* parasite load in liver overlaps with *Ir2* (28) and *Ltr2* (23) that control parasite load in liver after infection with *L. donovani* and *L. tropica*, respectively. Thus, similarly as *H2* or *Slc11a1*, some parasite-controlling *Lmr* loci might affect several pathogens, probably reflecting immune responses effective against groups of infectious agents.

### Relationship Between Control of Parasite Load, Organ Pathology, and Systemic Immune Response

We assessed relationship between parasite control, organ pathology, and systemic immune response (Table 11) and found a large heterogeneity in effects of controlling loci. Moreover, control of parasite load was linked with control of organ pathology in some loci, but not in others. Some loci, such as *Lmr13* and *Lmr14* carried by CcS-16, and *Lmr15* and *Lmr24* carried by CcS-9 controlled both parasite loads in organs, organ pathology and systemic immune response: *Lmr13* determined parasite load in spleen and liver, skin lesions (40), IgE in serum (44), TNFα in serum (45); *Lmr14* (CcS-16) influenced parasite load in liver (suggestive linkage), splenomegaly, hepatomegaly (40), IgE (44), IFNγ, IL-12, and TNFα in serum, and spontaneous proliferation of splenocytes from infected mice (45); *Lmr15* (CcS-9) controlled parasite load in lymph nodes and in liver, infiltration of eosinophils into lymph nodes (33), skin lesions, hepatomegaly, IL-4 and IgE in serum; and *Lmr24* controlled parasite load in lymph nodes, skin lesions, splenomegaly and IL-4, IL-10, and IFNγ in serum). *Lmr14* carried by CcS-9 determined parasite load in lymph nodes and in liver and systemic immune response (IL-13 in serum), but no organ pathology. *Lmr15* carried by CcS-16 did not influence parasite load, but controls hepatomegaly (40) and IFNγ in serum (45). Loci *Lmr14* and *Lmr15* might represent clusters of genes, their STS-derived segments in CcS-9 and CcS-16 overlap, but are not identical and they controlled different combinations of parameters in these strains (Tables 11A,B). Loci *Lmr4, Lmr11*, and *Lmr27* controlled parasite load in organs, but neither organ pathology nor systemic immune response (Table 11). Locus *Lmr25* controls parasite load in lymph nodes, infiltration of eosinophils to the lymph nodes, but no skin or visceral pathology. Locus *Lmr18* influences only skin lesions. *Lmr26* controls only infiltration of eosinophils to the lymph nodes (33), and locus *Lmr5* carried by CcS-16, *Lmr12* and *Lmr19* control only systemic immune response to *L. major* (44, 45).

We have observed a discrepancy between genes controlling parasites in organs and genes controlling splenomegaly and hepatomegaly. In the strain CcS-9 parasite load in the liver was controlled by the loci *Lmr14, Lmr15*, and *Lmr27*, but only one of them, *Lmr15*, was involved in control of hepatomegaly. Similarly, in the strain CcS-16, parasite load in liver were controlled by the loci *Lmr13* and *Lmr14* (suggestive linkage), whereas hepatomegaly is determined by *Lmr14* and *Lmr15*. In the strain CcS-9 we did not detect loci controlling parasite load in spleen (see above); splenomegaly was controlled by loci *Lmr15* and *Lmr24*. In the strain CcS-16 parasite load in spleen was controlled by *Lmr11* and *Lmr13*, but none of these loci was involved in control of splenomegaly, which was determined by *Lmr14*. The difference between the genes controlling parasite load and spread to an organ and those controlling pathology of this organ reflects the multiple facets of interaction between pathogen and host.

In visceral leishmaniasis, *Leishmania* amastigotes exist and proliferate in the mononuclear phagocytic system, especially spleen, liver and bone marrow. The response of immune system might lead either to parasite clearance or to unproductive inflammation resulting in organ hyperplasia (48, 49). These processes involve multiple steps that are regulated by different genes. The presented data are the first step in creating genetic model of visceralization, the most important pathology caused by *Leishmania* parasites, by identification the loci that control invasion of parasites and loci controlling the inflammatory response of the host.

### Sex-Dependent Control of Parasite Load

Control of parasite load in inguinal lymph nodes is in many cases sex dependent. In the strain CcS-9, *Lmr4*, and *Lmr27* controlled parasite load in males only; *Lmr15* controlled parasite load both in females and males, but with the opposite direction: BALB/c (C) allele is linked with higher parasite load in females and lower parasite load in males. Sex has been found to influence susceptibility to many diseases, including leishmaniasis (50, 51) and genes controlling infections that are sex dependent have been observed with other infectious agents such as viruses (52, 53), bacteria (54), parasites (33, 55), fungi (56), and helminths (57). All the above mentioned loci are localized on autosomal chromosomes and thus are shared by both sexes, but the regulatory genome is sexually dimorphic (58). The regulatory genome includes steroid hormones responsive elements (59), sex-specific micro-RNA (60) and sexually dimorphic DNA methylation patterns, which vary significantly from tissue to tissue (61). Generally, the sex differences have complex functional structure and their explanation requires future molecular identification of the responsible genes.

Further studies are needed to show how these mechanisms influence frequent sex differences in human leishmaniasis. Human epidemiological studies demonstrated that women are less likely to develop leishmaniasis while men tend to be more susceptible (62), although there are exceptions (50, 63). Some epidemiological studies reported no significant sex differences in registered cases of cutaneous leishmaniasis caused by *L. tropica* (64) and *L. major* (65) between men and women. However, other studies revealed in male patients a higher incidence of cutaneous leishmaniasis caused by *L. major* and *L. tropica* (66, 67), *L. major* only (68), and also by *L. guyanensis* (69). Men were also more susceptible to visceral infection caused by *L. donovani* (70–72), *L. infantum* (73–76). As an exception to this general trend, the study in Afghanistan found that females developed more lesions and scars after *L. tropica* infection (77).

Our data do not allow conclusion of influence of sex on parasite load in organs of the strain CcS-16. We tested only females in this strain, because in previous experiments, they did not exhibit sex differences in lesion size (34), however CcS-16 might exhibit sex differences in parasite load.

## Conclusions

The present study was focused on the genetic basis of one of the most important parameters of *L. major* caused leishmaniasis—parasite load in target organs. The study used a hypothesis-free experimental approach and recombinant congenic mouse strains to perform genome wide mapping of a complex system of genes that regulate dissemination of the parasite inside a mammalian organism and form a network-like structure (Figure 3). Host genes controlling *L. major* revealed a wide variety of heterogeneous effects that included distinct organ-specific control, single-gene effects, gene-gene interactions and sex dependent control. The presented results contribute to the understanding of genetic aspects of leishmaniasis. Mapping of these genes and subsequent identification of prospective candidate genes will allow their functional analysis. In addition, the obtained information allows making focused tests of human orthologous genes for their possible role in leishmaniasis and to elucidate pathogenesis and visceralization in individual patients.

## Ethics Statement

The experiments were performed in accordance with the European Union guidelines for work with animals under the Policy of Animal Protection Law (No.246/1992), and also with the regulations of the Ministry of Agriculture of the Czech Republic (No.207/2004). The experiments were approved by the Institutional Animal Care Committee of the Institute of Molecular Genetics AS CR and by Departmental Expert Committee for the Approval of Projects of Experiments on Animals of the Academy of Sciences of the Czech Republic (permissions 12/2002 and 89/2013).

## Author Contributions

TK and MČ conceived the study, performed experiments, interpreted the data and wrote the manuscript. VV performed experiments, analyzed the data and contributed to the writing of the manuscript. YS, HH, and MS performed the experiments. PD analyzed the data and contributed to the writing of the manuscript. ML conceived the study, interpreted data and wrote the manuscript. All authors reviewed the manuscript.

### Conflict of Interest Statement

The authors declare that the research was conducted in the absence of any commercial or financial relationships that could be construed as a potential conflict of interest.

## Abbreviations

BALB: (Bagg and Albino) a standard inbred mouse strain
CcS/Dem: BALB/c-c-STS/Dem (recombinant congenic strain)
*Dice1*.2: determination of interleukin 4 commitment 1b
*H2*: histocompatibility-2
IL: interleukin
IFNγ: interferon gamma
IgE: immunoglobulin E
*Lmr*: *Leishmania major* response
*Ltr*: *Leishmania tropica* response
PCR-ELISA: polymerase chain reaction enzyme linked immunosorbent assay
QTL: quantitative trait locus
RC: recombinant congenic
*Slc11a1*, solute carrier family 11 (proton-coupled divalent metal ion transporters): member 1
SNB-9: saline-neopeptone-blood-9
STS: an inbred mouse strain of Swiss origin.
